# Double-Cross-Linked Networks Based on Methacryloyl Mucin

**DOI:** 10.3390/polym13111706

**Published:** 2021-05-23

**Authors:** Elena Olăreț, Brîndușa Bălănucă, Andra Mihaela Onaș, Jana Ghițman, Horia Iovu, Izabela-Cristina Stancu, Andrada Serafim

**Affiliations:** 1 Advanced Polymer Materials Group, University Politehnica of Bucharest, 1–7 Ghe. Polizu Street, 011061 Bucharest, Romania; olaretelena@gmail.com (E.O.); brindusa.balanuca@yahoo.com (B.B.); andra_mihaela.onas@upb.ro (A.M.O.); jana.ghitman@upb.ro (J.G.); horia.iovu@upb.ro (H.I.); izabela.stancu@upb.ro (I.-C.S.); 2 Department of Organic Chemistry Costin Nenitescu, University Politehnica of Bucharest, 1–7 Ghe. Polizu Street, 011061 Bucharest, Romania; 3 Academy of Romanian Scientists, 54 Splaiul Independentei, 050094 Bucharest, Romania; 4 Faculty of Medical Engineering, University Politehnica of Bucharest, 1–7 Ghe. Polizu Street, 011061 Bucharest, Romania

**Keywords:** porcine gastric mucin, methacryloyl mucin, double-cross-linked networks, circular dichroism, mechanical characterization

## Abstract

Mucin is a glycoprotein with proven potential in the biomaterials field, but its use is still underexploited for such applications. The present work aims to produce a synthesis of methacryloyl mucin single-network (SN) hydrogels and their double-cross-linked-network (DCN) counterparts. Following the synthesis of the mucin methacryloyl derivative, various SN hydrogels are prepared through the photopolymerization of methacrylate bonds, using reaction media with different pH values. The SN hydrogels are converted into DCN systems via supplementary cross-linking in tannic acid aqueous solution. The chemical modification of mucin is described, and the obtained product is characterized; the structural modification of mucin is assessed through FTIR spectroscopy, and the circular dichroism and the isoelectric point of methacryloyl mucin is evaluated. The affinity for aqueous media of both SN and DCN hydrogels is estimated, and the mechanical properties of the systems are assessed, both at macroscale through uniaxial compression and rheology tests and also at microscale through nanoindentation tests.

## 1. Introduction

Mucin represents a major component of mucus, which covers several tissues (e.g., nose, eyes, gastrointestinal and respiratory tract, articular joins, etc.) with the aim of protecting them from the adsorption of unwanted molecules, reducing friction, preventing wear [1,2] or even autodigestion, in the case of gastric mucin [3]. Regardless of the covered tissue, mucus is secreted and degraded continuously, and most nanospecies are unable to penetrate it, being ultimately covered in a thin mucus layer before reaching the epithelial tissue [4]. Generally speaking, mucins contain a long polypeptide backbone rich in proline, threonine, and serine (PTS domain), on which dense brushes of carbohydrate chains are grafted [3,5,6]. Mucins are large compounds with a molecular weight of up to 20 MDa, and while the protein represents only about 20% of their molecular weight, the rest is made up of *O*-linked and *N*-linked oligosaccharides [1,6,7]. The composition and structure of mucins depend on their origin and are mirrored in slightly different behavior. For example, Madsen et al. demonstrated that while both bovine submaxillary mucin (BSM) and porcine gastric mucin (PGM) form hydrated films on hydrophobic surfaces, the adsorbed layers have different mechanical properties (BSM leads to elastic films, while those formed by the PGM are viscous), and the authors attributed this behavior to the structural differences between the two macromolecules [8]. Although mucin is pricier than other natural polymers such as collagen, gelatin, chitosan, and alginate, it is considered a material of interest for a variety of applications in the biomedical field. Since commercial mucins do not exhibit the required features for the aimed applications (e.g., virus inhibition, hydrogel formation, etc.) [7], these products are often purified through chromatography [5,9,10], thus increasing the actual price of the starting material. Nevertheless, the complexity of their structure proved to be appealing, and mucins have been studied for their use as carriers for bioactive species [11,12], coatings with improved tribological performance [13,14,15], and antifouling properties [16,17,18,19]. Furthermore, to obtain chemically cross-linked stable hydrogels, protocols describing the modification of other natural macromolecules (such as gelatin [20,21] or chitosan [22,23]) have been used to modify mucin, and they allow for its subsequent polymerization in a manner similar to that performed for synthetic polymers. The direct reaction of mucin with methacrylic anhydride has been previously exploited in order to obtain systems with controlled drugs release properties [24,25]. Mucin’s methacryloyl derivative was also used as coating on polypropylene meshes for ventral hernia repair, and its potential as a bioactivator was proven through cellular tests using murine fibroblast cells [26]. However, as far as the authors of the present study are aware, the ability of methacryloyl mucin (MuMA) to form double-cross-linked network (DCN) systems has not yet been explored. 

Typically, double-network hydrogels contain simultaneously a tough but brittle network formed through covalent bonds and a secondary weak and ductile network obtained through physical interactions [27,28,29]. This combination results in a soft hydrogel with superior mechanical properties when compared to their single-network (SN) system counterparts [30] and is increasingly used in developing biomaterials with applications in substitutes for load-bearing soft tissues such as cartilage or muscles [27].

This work presents a two-step procedure of obtaining DCN systems based on methacryloyl-modified PGM by (1) covalent bonding of MuMA methacrylate groups through radical photopolymerization, leading to SN hydrogels; and (2) additional hydrogen bonds (H-bonds) formed between the functional groups of MuMA and tannic acid (TA).

Considering the well-researched influence of a medium’s pH on mucin behavior [31,32,33], in this work, hydrogels are prepared using media with different pH values. The synthesized SN systems are further incubated in a TA solution, and the properties of the obtained DCN hydrogels are compared with those of the SN counterparts. The efficiency of the photo-induced polymerization leading to SN hydrogels is assessed, and the affinity for aqueous media of all synthesized systems is evaluated. The mechanical properties of the obtained systems are assessed at both macro- and microscales. In addition, the study presents a characterization of the modified protein, using for the first time—in addition to Fourier-transformed infrared—the methods of circular dichroism spectroscopy and dynamic light scattering. This work sustains the idea that commercial PGM modified through a relatively easy and cost-efficient protocol may be used as a primary constituent in the synthesis of DCN hydrogels with applications in the field of biomaterials for load-bearing soft tissue.

## 2. Materials and Methods

All materials were purchased from Sigma-Aldrich (St. Louis, MI, USA) and used without any purification. Porcine gastric mucin type III (PGM), methacrylic anhydride (MA), carbonate-bicarbonate buffer (CB), phosphate buffer saline (PBS), 2-(N-Morpholino), ethanesulfonic acid hydrate (MES), and tannic acid (TA) were used in this experimental procedure. Here, 1-[4-(2-Hydroxyethoxy)-phenyl]-2-hydroxy-2-methyl-1-propane-1-one (Irgacure 2959) was used as a 10% ethanolic solution. HCl and NaOH were used as 1 mol/L solutions as pH adjusters. Double-distilled water (DDW) was used throughout the experiments.

### 2.1. Modification and Characterization of Natural Macromolecules 

#### 2.1.1. Modification of Commercial Porcine Gastric Mucin 

Mucin was modified through direct reaction with methacrylic anhydride (MA) by adapting the protocol reported in [24,25]. In brief, 1% *w*/*v* porcine gastric mucin (PGM) solution in a carbonate-bicarbonate (CB) buffer (0.05 mol/L) (pH 8) was cooled in an ice bath, followed by an addition of MA to a final concentration of 0.5% *w*/*v*. The reaction was kept under continuous stirring, at 4 °C overnight. The reaction mixture was dialyzed against double-distilled water (DDW), using MWCO 12–14 kDa dialysis membranes (Spectrum™ Labs Spectra/Por) for 24 h with 3 changes of water, at room temperature. The obtained mixture was freeze-dried (48 h, −80 °C) and kept at −20 °C until later use for the hydrogel synthesis. 

#### 2.1.2. Fourier Transform Infrared (FTIR) Spectroscopy

FTIR spectroscopy was employed to assess the efficiency of the PGM modification. To this end, analyses were performed using a Vertex 70 Brucker FTIR spectrometer, equipped with an attenuated total reflectance (ATR) accessory. All FTIR spectra were recorded at room temperature, using 32 scans in the 600−4000 cm^−1^ wavenumber region, with a resolution of 4 cm^−1^. Fourier self-deconvolution was done using Omnic software in the spectral region of 1500–1750 cm^−1^ and also in 850–1200 cm^−1^. This computational tool was used to elucidate these wavelength intervals of interest, where the overlapped bands were detected in a separate manner and the important changes in the chemical structure of the modified mucin could be identified. This method was also employed to evaluate the formation of DCN systems, which is later presented.

#### 2.1.3. Circular Dichroism (CD) Spectroscopy

CD spectroscopy was performed to characterize the secondary and tertiary structures of MuMA. The spectral results were registered using the JASCO J-1500 circular dichroism spectrophotometer equipped with a Peltier cell holder unit for precise temperature control. Data were collected in the wavelength range 176–350 nm using a 100 nm/min scanning speed, at a 1 nm bandwidth. A 0.025 nm data pitch and 3 accumulations for each analysis were used. The samples were analyzed as 0.5 mg/mL solutions in a 1 mm path-length quartz rectangular cell.

#### 2.1.4. Zeta Potential

The zeta potential (ζ) of PMG and MuMA was determined in order to evaluate the modification of the isoelectric point (IP) of the modified protein when compared to the pristine commercial product. The IP is defined as the pH value at which the zeta potential value is zero, implying no electric charge on the surface of molecule [34]. The zeta potential of PMG and MuMA was measured using a Zetasizer Nano-ZS (Malvern Instruments Ltd., United Kingdom) equipped with a He–Ne linear polarized laser operating at a wavelength of λ = 632.8 nm and an angle of 13°. The equipment determines the electrophoretic mobility of colloids, employing the principle of laser Doppler velocimetry which is converted to a ζ-potential using the Helmholtz–Smoluchowski equation [35]. For these measurements, 1 mg/mL solutions of PMG and of MuMA, with pH values ranging from 1 to 6, were prepared in DDW, loaded in transparent rectangle cuvette with electrodes at each end and subsequently placed in the equipment where an electric field was applied to the cell. The pH of the tested samples was adjusted using 1 mol/L HCl solution and NaOH 1 mol/L solution, both prepared using DDW. For each sample, 3 measurements with 20 successive cycles at 25 °C were performed.

### 2.2. Synthesis of MuMA-Based Networks 

Hydrogels consisting of 10% *w*/*v* MuMA in three different media (i.e., PBS, MES, and CB) were synthetized through photopolymerization. The corresponding amount of MuMA was gradually added in each of the previously mentioned media, followed by 2 h 30 min of vigorous stirring at 4 °C. 0.1% *w*/*w* (with respect to MuMA content), after which Irgacure 2959 was added. After homogenization, the reaction mixtures were poured into Petri dishes, followed by UV exposure (30 min at 312 nm wavelength using UV transilluminator ECX-F26). As-obtained samples of each composition were dried and preserved for the evaluation of polymerization efficiency through gel fraction assessment. The rest of the synthesized hydrogels were rinsed with DDW and cut into suitable geometries for further evaluation. Additionally, samples from each hydrogel composition were immersed in 10% TA solution in DDW for 24 h to form the secondary network, while their corresponding control samples were incubated 24 h in DDW. Hydrogels composition and codes are presented in Table 1.

To avoid any interference from the dispersion media, the hydrogels subjected to FTIR measurements were synthesized in DDW following the same procedure as previously described. 

### 2.3. Gel Fraction Evaluation 

The efficiency of the network-forming polymerization was correlated with the polymerization reaction media through a gel fraction (*GF*, %) assessment. Briefly, samples of each synthesized system were dried (at 37 °C for 24 h) immediately after synthesis. Following the complete evaporation of the solvent, the samples were weighted (*w*_0_) and subsequently washed thoroughly in DDW for 24 h at 37 °C, then were dried and weighted again (*w_f_*). GF was assessed through Equation (1) [36]. The measurements were performed in triplicate.

(1) $$GF,\;\%=\frac{w_{f}}{w_{0}}\;\times \;100$$

The GF analysis aimed exclusively at the assessment of the polymerization efficiency and was performed only on the SCN systems. The DCNs were obtained through the immersion of the synthesized hydrogels in 10% TA aqueous solution for a period of 24 h, and it was expected that any unbounded protein chains would be dispersed in the incubation medium, and thus the GF analysis would result in inaccurate data.

### 2.4. Swelling Ability

The affinity for aqueous media of the MuMA and MuMA_TA systems was investigated through a conventional gravimetric method. In this respect, dried samples of each composition were weighed (*w*_0_), immersed in DDW at 37 °C, removed from the incubation medium, blotted with filter paper, and weighted at pre-established time points (*w_t_*). All measurements were performed in triplicate, and the swelling content (SC) was determined, as in Equation (2). A SC–time graph was plotted, and the swelling profile of the synthesized systems was constructed [37].

(2) $$SC,g/g=\frac{w_{t}-w_{0}}{w_{0}}$$

In addition, the equilibrium water content (EWC, %) was computed as reported previously [36] for both SN and DCN systems, using Equation (3).

(3) $$EWC,\;\%=\frac{w_{max}-w_{0}}{w_{max}}\times 100$$

where *w_max_* represents the weight of the hydrogel at swelling equilibrium.

### 2.5. Mechanical Investigations 

Mechanical assessments were performed to characterize the synthesized hydrogels at both macro- and microscales. To this end, uniaxial compressions were carried out to estimate the reinforcing role of TA in the DCN hydrogels, and rheology tests offered information about the storage and loss moduli of the bulk samples, while nanoindentation provided insights regarding the correlation local microstructure–mechanical behavior. 

#### 2.5.1. Uniaxial Compressions 

The synthesized systems were subjected to compression tests using a CT3 Texture Analyzer (Brookfield) equipped with a 4500 g cell. Cylindrical samples (d = 5 mm, h = 2 mm) were compressed up to 50% deformation, and the strain–stress curves of the compositions were plotted. 

#### 2.5.2. Rheological Tests

The investigation of the rheologic behavior was performed through dynamic oscillatory measurements using a Kinexus Pro rheometer equipped with a Peltier element for precise temperature control, performed at 25 °C. Samples of hydrogel hydrated at equilibrium (d = 20 mm, h = 1.5 mm) were placed on the bottom plate of the rheometer, and parallel-plate geometry was used. Sand paper was used on the plates to avoid slipping. Dehydration was prevented using a water lock. The linear viscous region was established through amplitude sweep measurements, and subsequently a frequency sweep analysis was performed in the frequency range of 0.01–100 Hz at a constant stress value of 10 Pa. The storage and loss moduli (G’ and G”, respectively) were plotted in a logarithm graph.

#### 2.5.3. Nanoindentation 

Local mechanical characterization was carried out through nanoindentation using the Nano Indenter G200 system supplied with a DCM II head and CSM option (KLA Instruments). The shear storage modulus (G’) and loss modulus (G”) were obtained using a cylindrical flat punch diamond tip with a face diameter of 100 µm. Four different sites were tested for each hydrogel sample, while ensuring a minimum distance of 500 µm between them. The testing frequency was set to 10 Hz. Poisson’s ratio was set to 0.5 (as it is typical for gels), and the oscillation amplitude (in sample) was set to 500 nm. Pretest compression was kept at 5% of the punch diameter.

## 3. Results and Discussions

### 3.1. Synthesis and Characterization of PGM Methacryloyl Derivative 

The reaction of mucin with methacrylic anhydride was first described by Duffy et al., who confirmed the modification of bovine submaxillary mucin by FTIR spectroscopy and the ability of the macromolecule to form stable hydrogels [25]. Following the same modification protocol, our work group previously reported the use of porcine gastric mucin (PGM) methacryloyl derivative (MuMA) in hybrid systems with applications in wound treatment [24] and as coating for the bioactivation of polypropylene meshes for ventral hernia repair [26]. Herein, we used a slightly modified version of the previous protocols, namely, a carbonate buffer (CB) was used as the reaction medium for the modification. The pH was monitored throughout the reaction, and no additional measures were required to maintain the pH at 8. The circular dichroism (CD) spectra registered for PGM in the CB confirm that at pH 10, the PGM molecule was less folded when compared to the spectra registered for PGM in DDW (pH 6) (Figure S1). The formation of a small shoulder-like peak at approximately 185 nm for PGM in CB was noticed. This is characteristic for a better exposure of lateral groups to the surrounding medium and therefore a better unfolding of the PGM structure. Moreover, a small decrease in the intensity of the peak at approximately 190 nm suggests the unfolding of the polypeptide chain for higher pH medium. As a consequence, it is expected that the NH_2_ groups are more exposed, allowing for the reaction with the anhydride. 

**Figure 1:**
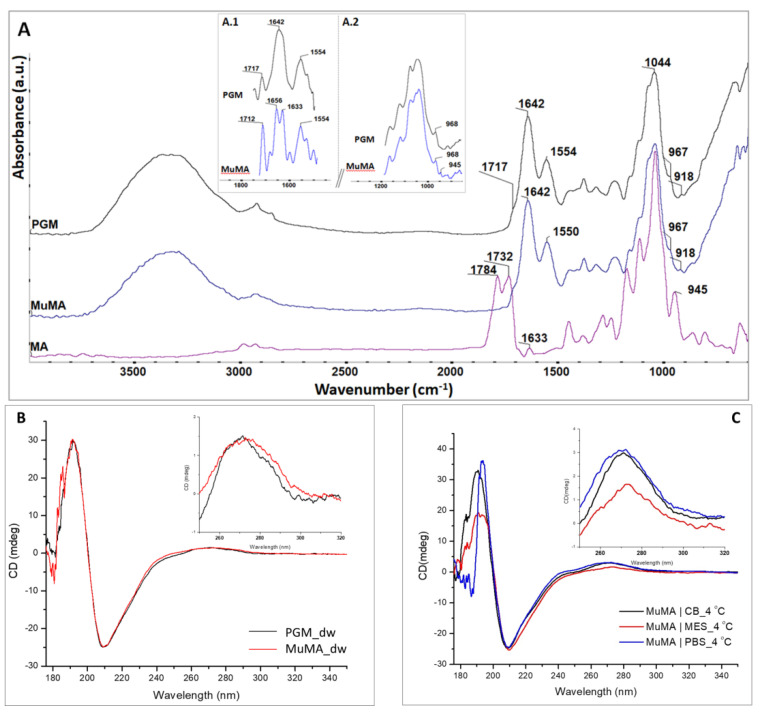
(**A**) FTIR spectra of PGM (**black**), MuMA (**blue**), and MA (**purple**); inset A.1: deconvoluted spectra of PGM and MuMA in the wavenumber region 1500–1750 cm^−1^; inset A.2: deconvoluted spectra of PGM and MuMA in the wavenumber region 850–1200 cm^−1^; (**B**) CD spectra of PGM and MuMA; inset: magnification in the 250–320 nm region; (**C**) CD spectra of MuMA|MES, MuMA|PBS, and MuMA|CB at 4 °C; inset: magnification in the 250–320 nm region.

Following the synthesis and purification of MuMA, spectral analyses were employed to characterize the freeze-dried product. The registered ATR-FTIR spectra for native PGM, MA, and MuMA are presented in Figure 1A. The FTIR spectrum of MuMA shows all characteristic signals of a protein [36]: a broad peak in the region 3100–3700 cm^−1^ attributed to overlapped N–H and O–H stretching vibration, an amide I typical signal at 1642 cm^−1^, and a peak attributed to amide II that slightly shifted from 1554 cm^−1^ in PGM to 1550 cm^−1^ in MuMA. In order to gain a better visualization of the specific vibration bands from MA, a deconvolution process of the MuMA spectrum was performed in the 1500–1750 cm^−1^ and 850–1200 cm^−1^ regions (Figure 1A, inset A.1 and A.2, respectively). The outcome reveals the presence of two peaks, i.e., around 1633 cm^−1^ and 945 cm^−1^, which were assigned to the stretching vibration of –C=C– bonds and the deformation of =CH_2_ bonds, respectively, and were specific bands for the new grafted methacrylate groups [38,39]. 

The successful methacrylation was also confirmed through CD spectroscopy. As depicted in Figure 1B, the spectrum registered for MuMA presents a prominent shoulder around 182 nm attributed to the n–σ* transition of the C=O groups introduced by the methacrylation process. Additionally, the CD results indicate that the methacrylation process does not lead to the denaturation of the polypeptidic chains, as the registered MuMA spectra indicate an unaltered tertiary structure of the methacryloyl derivative, similar to the one of the pristine protein. Moreover, CD spectroscopy indicated that the MA-modified product maintains the pH dependence of the pristine PGM. The spectra registered for MuMA|MES, MuMA|PBS, and MuMA|CB indicated that the methacryloyl derivative is most unfolded in an acidic medium. Figure 1C depicts the MuMA spectrum in each medium at 4 °C.

For the MuMA|MES CD spectra, a decrease of the positive contribution—as reflected by the magnitude of the peak—at around 190 nm may be noticed, which is associated to the π–π* perpendicular polarized transitions of helix-containing polypeptides, suggesting a less folded structure at pH 4. From the 250–320 nm region, we observed that the MuMA|CB sample exhibits the most folded tertiary structure of the polypeptidic chains, while for the MuMA|MES sample, a slight deviation of the 270 nm peak suggests a possible modification of the folded architecture, and its obvious decrease in intensity could be assigned to an unfolding of the macromolecular chains.

The CD spectra for MuMA samples in all three media were registered both at 4 and 25 °C (Figure S2). The results indicate that in MES, the polypeptide chains were more unfolded at 4 °C. There was a clear reduction in the positive contribution at approximately 190 nm for the MES spectra at low temperature when compared to ambient temperature (25 °C) caused by the unfolding of the molecule. Conversely, the spectra registered for MuMA in PBS showed that at 4 °C, the positive contribution around 195 nm increased, leading to a more tightly folded structure of the macromolecule when compared to ambient temperature spectral results. At high pH values, the arrangement of the macromolecular chains was not influenced by this parameter, as the spectra registered for MuMA in the CB is similar for the two investigated temperatures. 

Zeta potential measurements were also performed to characterize the methacryloyl derivative. Generally, it is known that zeta potential is a function of the surface charge of a colloid in solution as well as of the pH and ionic strength of the solution. In the pH range of 5–2.5, the zeta potential of both PMG and MuMA was negative due to the negatively charged carboxylic functionalities (–COO–) from the protein chains. The decrease of pH value below 2.5 led to a positive reversion of the zeta potential, exhibiting a cross point (IP value) at pH ≈ 2.1 for PMG and pH ≈1.1 for MuMA (Figure S3). In this pH range, the amino groups from protein chains were positively charged (–NH_3_^+^) and the zeta potential became positive. The results regarding the IP of the unmodified protein are in agreement with the findings of other research groups that have reported an IP value in the range of 2–3 [6,40].

The IP value of MuMA shifted to a lower pH value when compared to that of PMG. In the methacrylation reaction of PMG, the primary amino groups (–NH_2_) were mostly involved, covalently binding the methacrylic functionality on the protein chain. This led to the formation of MuMA and methacrylic acid as a byproduct that is easily eliminated from the system by dialysis. Thus, this pH shift might have originated in the increase of the carboxyl/amino groups’ ratio from the MuMA structure (considering that a part of the –NH_2_ functionalities are consumed in the methacrylation reaction). These results are in good agreement with the previously discussed FTIR data, indicating that some of the -NH_2_ groups were consumed during the reaction with the methacrylic anhydride.

### 3.2. Synthesis of the SN and DCN Systems 

Considering the pH dependency of MuMA, as indicated by the CD spectroscopy, the polymerization precursors were prepared using reaction media with different pH values, all above the IP of MuMA (approx. 1.1 as indicated by zeta potential investigations). As a consequence of this pH dependency, corroborated with the amphoteric character of the polymer due to the abundance of both positively and negatively charged groups (unmodified –NH_3_^+^ groups form the protein core, and –O– and –COO– form the carbohydrates side chains), it was expected that the reaction media of the polymerization process would imprint a certain orientation of the MuMA’s chains. 

**Figure 2:**
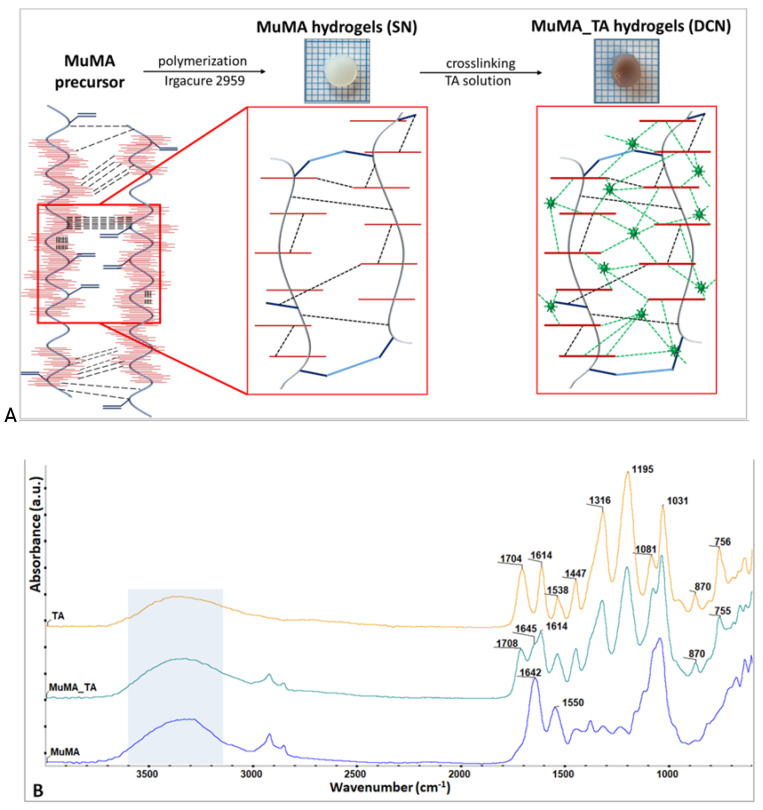
(**A**) Schematic representation of the synthesized SN and DCN systems (blue: “C–C” covalent bonds formed through the polymerization of “C=C” groups in MuMA; dashed black: intra- and intermolecular H-bonds in MuMA; dashed green: TA-MuMA H-bonds); (**B**) FTIR spectra of TA (**orange**), MuMA_TA (**teal**), and MuMA (**blue**).

The DCN systems were synthesized through a two-step procedure consisting of (1) the formation of SN systems through covalent bonds established through the bulk polymerization of the methacrylic groups of MuMA, followed by (2) the formation of an additional physical network based on H-bonds established through TA diffusion into the MuMA SN. While the first step of the process led to opaque, easy-to-handle, soft hydrogels, following the incubation in TA the hydrogels changed to a brownish color and exhibited a slight shrinkage with a more robust nature. A schematic representation of the synthesis procedure is shown in Figure 2A.

Given the numerous functional groups of MuMA, intra- and intermolecular H-bonds were formed in the hydrogel precursor prior to the photopolymerization of “C=C” bonds. SN formation led to new “C–C” covalent bonds, while TA treatment additionally strengthened the hydrogel with TA–MuMA H-bonds, resulting in DCN.

### 3.3. Assessment of the Polymerization Process 

Successful polymerization was confirmed through the GF analysis, based on gravimetric measurements and using Equation (1). The overall results indicate that the polymerization was successful for all compositions. The GF values that were computed for all three systems are in the range of 68–78%, indicating a successful polymerization process that led to insoluble, stable hydrogels regardless of the pH of the reaction media. However, slight differences between the analyzed SN systems were noticed (Table 2).

Considering that all systems incorporated the same solid content and were synthesized following the same protocol, the different values obtained for GF are attributed to the different pH values of the polymerization media, and they are in good agreement with the insights provided by CD spectroscopy, which indicated the highest GF for the system obtained in MES (in which MuMA exhibited the most unfolded structure at 4 °C). This behavior led to a better exposure of the methacrylic groups on the polypeptidic chains and thus the formation of covalent bonds in the presence of the photoinitiator. Conversely, the macromolecular chains are more folded, exposing fewer reaction centers in the systems in which PBS and CB were used, i.e., the dense “brush” of carbohydrates hindering the methacrylic groups and therefore leading to the formation of systems with lower GF values. The lowest GF value was registered for the systems obtained in PBS, indicating that the macromolecular chains were neither unfolded enough to sufficiently expose the methacrylic groups (as they were in MES) nor properly folded to form additional physical bonds (as this is probably the case with the systems synthesized in a CB). These findings are in agreement with the CD results, which show that the MuMA|PBS spectrum exhibited in the region of 190–210 nm a narrower peak of higher intensity when compared to the other registered spectra, confirming the distinct folding of the modified protein at pH 7.4 and 4 °C.

The GF analysis was not considered relevant for the DCN systems due to the relatively long period of incubation (24 h) in 10% TA aqueous solution. In these conditions, it was expected that any unbounded modified protein would be lost in the incubation medium, thus leading to incorrect results. 

The formation of DCN systems was firstly evaluated through FTIR spectroscopy; the corresponding spectra for TA, MuMA, and MuMA_TA are presented in Figure 2B. The MuMA_TA spectrum shows specific signals attributed to TA and also the MuMA matrix in 1600–1710 cm^−1^ region as follows: 1708 cm^−1^, ν_C=O_ and aromatic ν_C=C_ from TA; 1614 cm^−1^, ν_C–C_ and δ_C–H_ from the TA aromatic structure; and 1645 cm^−1^, amide I from long protein backbone of PGM. All other absorption bands in between the 600–1538 cm^−1^ wavenumber interval could be assigned to the specific bond vibrations from PGM and TA [14]. Intermolecular H-bonds formed between the –OH groups from TA and –COOH moieties from the MuMA-based polymerized structure during the incubation process led to the shape modification of the broad band at 3000–3600 cm^−1^. The TA spectrum indicates a more intense and broader O–H band at higher wavenumber values, due to the many hydroxyl groups from its structure while the O–H stretch band for MuMA slightly shifted to lower wavenumber values due to the great number of –COOH moieties from the protein region of macromolecule.

### 3.4. Investigation of the Water Uptake Potential of the Synthesized Systems 

One of the most exploited features of hydrogels is their water affinity, as these materials are able to absorb large quantities of aqueous media without losing their dimensional integrity [41]. In addition, the time required to reach swelling equilibrium is also extremely important. This characteristic is pivotal for several precise applications, such as drug delivery and the design of scaffolds for tissue regeneration and repair [42,43,44]. Our study established not only the amount of water absorbed by the two type of systems but also their swelling profile, as in the computed parameters, i.e., equilibrium water content, (EWC, %) and swelling content (SC, g/g), providing important insights regarding the time required for the synthesized systems to reach the maximum hydration and also the maximum amount of aqueous media they are able to uptake.

**Figure 3:**
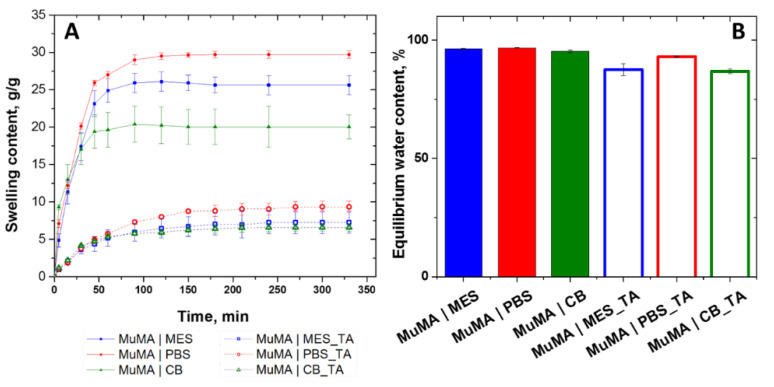
(**A**) Swelling kinetics representing the SC increase during hydration of SN and DCN systems; (**B**) equilibrium water content (EWC, %) computed for the SN and DCN hydrogels.

The swelling profile was represented as a function of time, and it indicated that there are major differences between the synthesized systems. As depicted in Figure 3A, the highest SC was registered for MuMA|PBS, which is in accordance with the GF study and thus confirming the formation of networks that allow for the uptake of large amounts of water. Conversely, the more tightly packed networks of MuMA|MES and MuMA|CB, for which a higher GF was obtained, led to slightly lower SC values. These differences are less pronounced in the case of the DCN systems, indicating that the formation of the supplementary network through incubation in TA is efficient for all systems, leading to more compact matrices. Moreover, the considerably lower values of SC registered for the DCN when compared to the SN counterparts confirm the formation of a second dense network, leading to systems with a decreased water uptake ability. Furthermore, the DCN systems require a longer period of time to reach swelling equilibrium (250 min) when compared to the SN systems (180 min) (Figure 3A). 

After swelling equilibrium was reached, EWC values were computed, which are represented in Figure 3B. Although there are no significant differences between the EWC of the SN systems, the data show that the formation of the secondary network had a different impact on the obtained DCN systems. The EWC computed for the systems that were synthesized in MES presented the highest difference between SN (96.24 ± 0.19) and DCN (87.47 ± 2.5), while the MuMA|PBS registered a value that is only 3.79% higher than MuMA|PBS_TA. This behavior is also a confirmation of the different orientation of the functional groups in MuMA structure during the photopolymerization process due to the pH value of the dispersion media, which ultimately leads to a more unfolded (in the case of MES) or folded (in the case of PBS and CB) structure. 

### 3.5. Evaluation of the Mechanical Behavior of the SN and DCN Systems

The hydrogels’ mechanical properties are generally considered weak, and thus strategies to enhance mechanical properties while preserving their unique characteristics are in continuous development [45]. Among these, the sequential chemical and physical cross-linking of natural hydrogels proved to be a valid method for obtaining materials with superior mechanical behavior [46]. The results registered following conventional uniaxial compression tests of MuMA_TA hydrogels demonstrated that the DCN exhibited an improved resistance at break when compared to their SN counterparts. The strain–stress curves (Figure S4) show that the SN systems broke around a strain of 30–40% but were able to undergo higher deformation. Oppositely, the DCN systems required a significantly higher stress to be deformed at the same strain value, and this behavior correlated to the lower EWC and SC values. Furthermore, these data confirm that the interactions between the MuMA-based SN and TA significantly improved the materials’ resistance to deformation.

The storage modulus and the loss modulus of the composition were evaluated at macroscale through rheology tests performed on equilibrium-swollen samples. While the storage modulus (G’) provides information regarding the elasticity of the material due to the ability to store energy, the loss modulus provides insights into the ability to dissipate energy, and it is a measure of the hydrogel’s viscous behavior [47].

**Figure 4:**
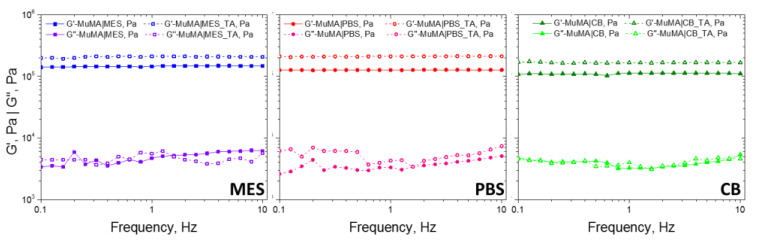
The rheological behavior of control samples and TA-treated samples.

The results show that elastic behavior is predominant in both SN and DCN hydrogels, with G’ higher than G”. In addition, G’ is independent of the frequency value in the studied interval, which shows good stability. As presented in Figure 4, regardless of the pH value of the polymerization medium, the formation of the supplementary network with TA led to a decrease of elasticity. Although the G’ value increased slightly, it is interesting that the G” value remained unchanged in the DCN systems, when compared with the SN ones. This detail suggests that the presence of the supplementary cross-linking of the hydrogel brought by the presence of H-bonds was not accompanied by the immobilization of any chain parts. The formation of the first network through covalent bonds led to soft yet brittle materials, while further incubating these materials in TA aqueous solution led to more robust systems that were able to undergo higher deformations without breaking under stress.

**Figure 5:**
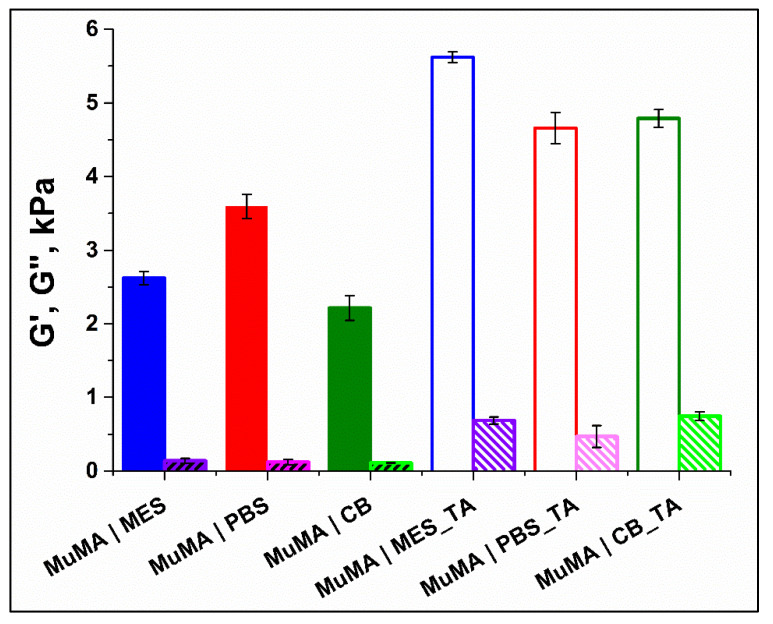
Microscale storage modulus (G’, simple bars) and loss modulus (G”, patterned bars) as obtained from instrumented indentation tests.

In addition to the macroscale mechanical properties of the synthesized materials, the study also offers important insights regarding their properties at microscale, as obtained through nanoindentation tests. Surface mechanical properties are of great interest when considering biomedical applications since they influence the cellular behavior [48]. As mentioned by Youssefian et al. [49], H-bonds act as springs between atoms and have a significant influence on polymer chains’ arrangement and on their elastic behavior. Thus, the storage and the loss moduli of equilibrium-swollen hydrogels were evaluated also at microscale through dynamic instrumented indentation tests (Figure 5). Overall, the storage moduli increased after SN incubation in TA, suggesting the surface reinforcing of the hydrogels. Such behavior may be assigned to MuMA–TA H-bonds formation, resulting in DCN. Furthermore, the surface elasticity placed the SN hydrogels in the order of MuMA|PBS < MuMA|MES < MuMA|CB, and TA treatment changed this pattern to MuMA|MES_TA < MuMA|CB_TA < MuMA|PBS_TA. TA exhibited a lower influence on MuMA|PBS (29.58% increase). For MuMA|CB, a doubling of the storage modulus was observed after TA incubation (97.85% increase). The highest increase in storage modulus after TA treatment was recorded for MuMA|MES_TA (114.45% increase). This is consistent with the CD results, where MuMA|MES presented the most unfolded structure, and therefore it was expected that this composition provides the most available sites to H-bond formation and hence a more pronounced TA-cross-linking effect

## 4. Conclusions

The successful synthesis of DCNs based on methacryloyl-modified porcine gastric mucin was demonstrated in three media with pH values representative for acid, neutral, and alkaline conditions. The study highlighted the importance of the pH value of the initial dispersion media on the folding of MuMA, as mirrored on the water uptake ability and the mechanical properties of the SN systems. Furthermore, our findings showed that the supplementary cross-linking with polyphenol allows an additional adjustment of the materials’ properties, both at the macro- and microscales. The conventional mechanical tests showed that TA cross-linking led to more robust systems that require additional effort to be deformed under stress. The surface mechanical properties indicated that the most significant influence of the supplementary cross-linking was exhibited on the MuMA|MES hydrogels, which doubled their storage modulus. Overall, the results demonstrated the capacity of MuMA to form DCN systems that have the ability to uptake large amounts of aqueous media without losing their integrity. The high water uptake ability and the macro- and microscale mechanical properties indicate that these systems may find applications in the field of tissue reconstruction and repair.

## Figures and Tables

**Table 1:** Hydrogel compositions and codes.

Type of System	Sample Code	Dispersion Medium	pH Value	Postsynthesis Incubation
Single network (SN)	MuMA|MES	MES	4.0	DDW, 24 h
	MuMA|PBS	PBS	7.4	
	MuMA|CB	CB	10.0	
Double cross-linked network (DCN)	MuMA|MES_TA	MES	4.0	10% TA aqueous solution, 24 h
	MuMA|PBS_TA	PBS	7.4	
	MuMA|CB_TA	CB	10.0	

**Table 2:** Gel fraction values registered for the MuMA-based SN systems.

Single-Network System	Gel Fraction (GF, %)
MuMA|MES	78.63 ± 1.04
MuMA|PBS	68.31 ± 0.72
MuMA|CB	75.62 ± 0.82

## Data Availability

The data presented in this study are available on request from the corresponding author.

## Supplementary Materials

The following are available online at https://www.mdpi.com/article/10.3390/polym13111706/s1; Figure S1: CD spectra registered for PGM in DDW and CB; the additional peak at 185 nm confirms a better exposure of the lateral groups of PGM at pH 10 (in CB) when compared to pH 6 (in DDW); Figure S2: CD spectra for MuMA in media with different pH values (MES at pH 4, PBS at pH 7.4, and CB at pH 10) registered at 25 °C and 4 °C; the results indicate that at 4 °C MES has the most unfolded structure, while PBS presents the most tightly packed structure; Figure S3: Graphical representation of the zeta potential measurements performed for the native mucin (PGM) and the methacryloyl derivative (MuMA). The results indicate that the modification with methacrylic anhydride decreases the isoelectric point from ≈2.1 (PGM) to ≈1.1 (MuMA); Figure S4: Stress–strain curves for both SN (solid line) and DCN (dash line) systems.
