# Supplementary material for: Double-Cross-Linked Networks Based on Methacryloyl Mucin
Source: Polymers (Basel). 2021 May 23;13(11):1706. doi: 10.3390/polym13111706 (PMC8197136; doi:10.3390/polym13111706)
Supplement: Supplementary file 1 [file polymers-13-01706-s001.zip › polymers-1214289-supplementary.pdf]

Supplementary Materials

Elena Olaret[1], Brindusa Balanuca[1,2], Andra Mihaela Onas[1], Jana Ghitman[1], Horia Iovu[1,3], Izabela-Cristina Stancu[1,4], Andrada Serafim[1*]

[1] Advanced Polymer Materials Group, University Politehnica of Bucharest, 1-7 Ghe. Polizu Street, 011061, Bucharest, Romania
[2] Department of Organic Chemistry Costin Nenitescu, University Politehnica of Bucharest, 1-7 Ghe. Polizu Street, 011061, Bucharest, Romania
[3] Academy of Romanian Scientists, 54 Splaiul Independentei, 050094, Bucharest, Romania
[4] Faculty of Medical Engineering, University Politehnica of Bucharest, 1-7 Ghe. Polizu Street, 011061 Bucharest, Romania
* Correspondence: andrada.serafim@gmail.com; andrada.serafim0810@upb.ro;

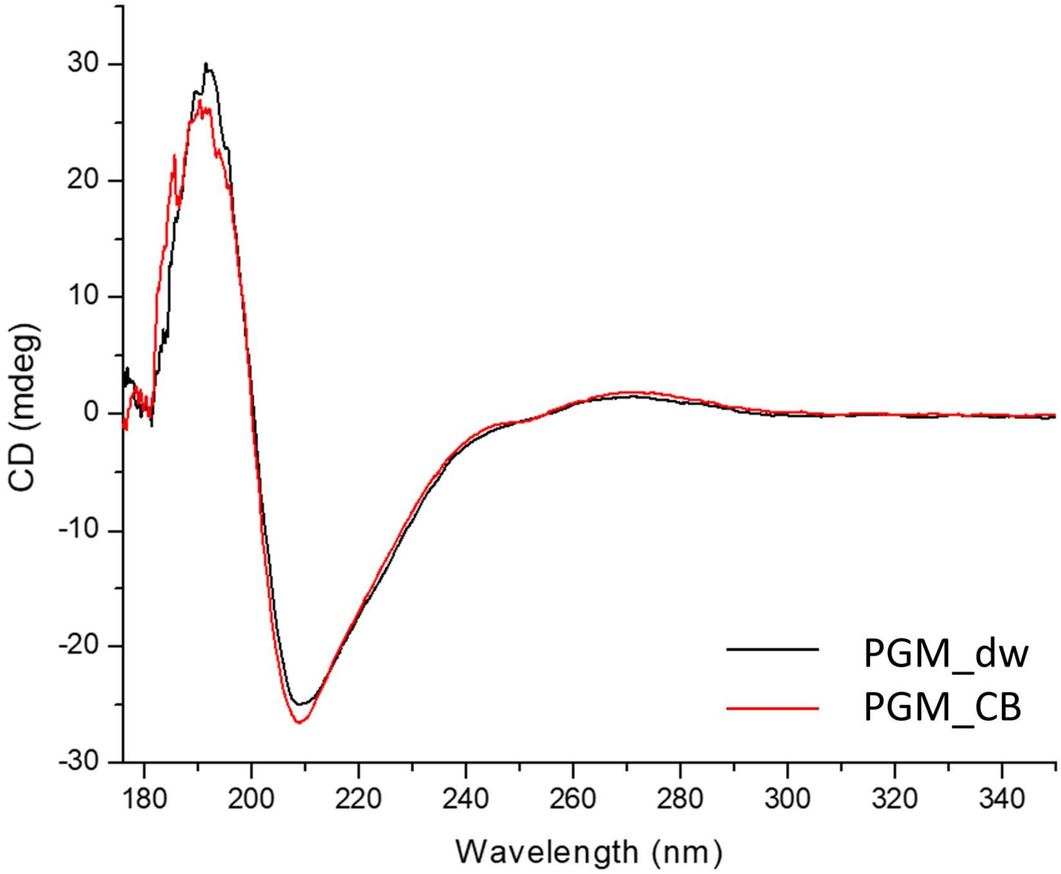

Figure S1. CD spectra registered for PGM in ddw and CB; the additional peak at 185 nm confirms a better exposure of the lateral groups of PGM at pH 10 (in CB) when compared to pH 6 (dw).

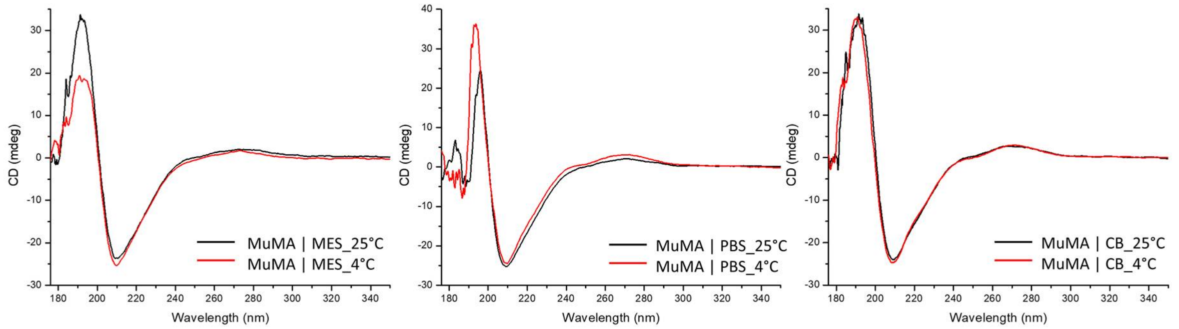

Figure S2. CD spectra for MuMA in media with different pH values (MES – pH 4, PBS – pH 7.4 and CB – pH 10) registered at 25 °C and 4 °C; the results indicate that at 4 °C MES has the most unfolded structure, while PBS presents a the most tightly packed structure.

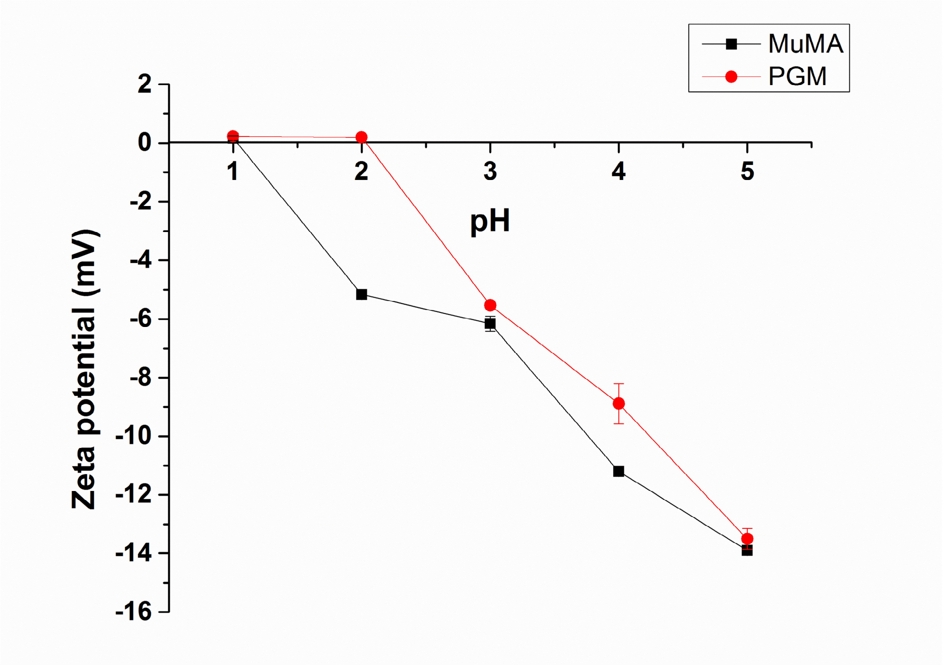

Figure S3. graphical representation of the zeta potential measurements performed for the native mucin (PGM) and the methacryloyl derivative (MuMA). The results indicate that the modifica-tion with methacrylic anhydride decreases the isoelectric point from~2.1 (PGM) to~1.1 (MuMA).

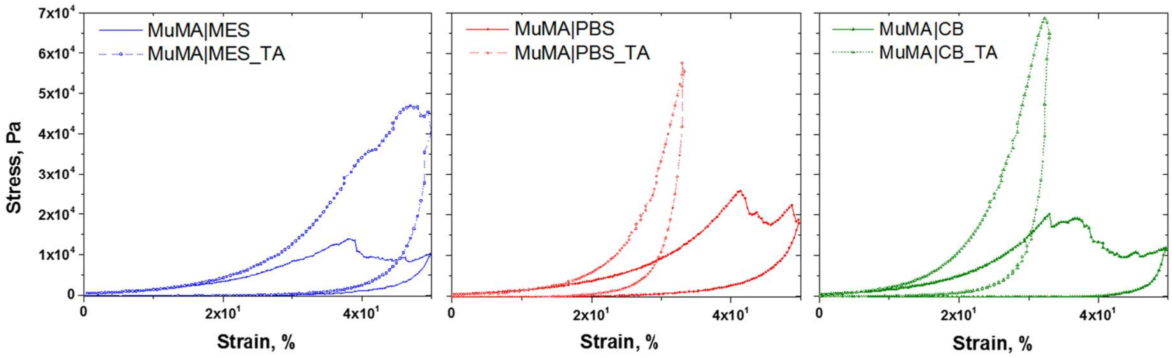

Figure S4. Stress-strain curves for both SN (solid line) and DCN systems (dash line).
